# The Medical Society of the State of New York

**Published:** 1896-03

**Authors:** 


**A Monthly Review of Medicine and Surgery.**

*EDITORS:*

THOMAS LOTHROP, M. D. - - WM. WARREN POTTER, M. D.

All communications, whether of a literary or business nature, should be addressed to the managing editor: 284 Franklin Street, Buffalo, N. Y.

Vol. XXXV. MARCH, 1896. No. 8.

### THE MEDICAL SOCIETY OF THE STATE OF NEW YORK.

THE 90th annual meeting of this society was held at Albany, January 28, 29 and 30, 1896. The meeting fell a week earlier this year, and thus avoided the crowd that generally gathers at Albany during the first week of February. There were several reasons that conspired to make this meeting of unusual interest. In the first place, it was held at Jermain Hall, which is an improvement over the old place of meeting. Again, interest was manifested in an address delivered by President Eliot of Harvard College. He chose a topic which is one of increasing importance at this time—namely, that of medical education. He contrasted that of thirty years ago with the methods of today, and after reviewing its various periods of evolution, finally reached the climax of his discourse in asseverating that medical education in the future would begin with preparation at the sixth year of age and continue until graduation, which would not ordinarily occur until the twenty-fifth. He laid great stress on the importance of early preparation, and asserted that physicians should interest themselves in the public schools to the end that the course of study there may be made of a nature to prepare children and youths for the later walks of life.

The report of the State Board of Medical Examiners contains interesting data. During the year there had been 603 examinations, of which license had been granted in 482 cases and there had been 171 rejections. The Eclectics rejected one, or 12 per cent.; the Homeopaths eight, or 12 per cent. and the State Board 162 or 27 per cent. Most of the states accept the New York State license, but owing to the fact that the standards are not as high in other states in all respects, it is not yet expedient for New York to exercise reciprocity in this matter.

Dr. A. Walter Suiter, of Herkimer, in discussing the Ainsworth instruction law that has been of late a matter of so much comment in the newspapers, and which provides for compulsory education with reference to alcohol and narcotics in the public schools, offered the following, which was adopted :

WHEREAS, This society is ever mindful of the evil results to individuals and to the community of the abuses of stimulants and narcotics and is ever ready to coöperate with and encourage anxious efforts to prevent such abuses ; and

WHEREAS, This society is familar with the recent attempt to force upon our public-school system the task of teaching our children the chemistry, the toxicology and the pathology of alcoholic stimulants and narcotic habits; and

WHEREAS, This society is in entire sympathy with the probable motives of the promoters of this law, but has profound consideration in its inexpediency ; therefore,

*Resolved*, That the committee on legislation is hereby instructed to bring about the repeal, or essential modification in the law relating to this subject.

We learn that it is highly probable that the law will be so changed as to make its provisions more in keeping with good sense and scientific knowledge.

Drs. George R. Fowler, of Brooklyn, and William C. Wey, of Elmira, were nominated to be State Medical Examiners to fill their own vacancies; and Drs. A. Walter Suiter and Willy Meyer were named as alternates.

The scientific part of the meeting was everything that could be desired, a full report of which has appeared in the weekly journals. An attempt was again made to change the place of meeting of the society on the plea that it would increase its efficiency, especially if it met in Buffalo or the western part of the state. This argument has been presented before, and the attempt has likewise been made to change the place of meeting, but it proved so disastrous to the interests of the society that it soon went back to the old way. The fact is, this society is a legal body, and is enabled to exercise much influence with reference to medical legislation and promote improvements in methods of medical education from the very fact that it meets in Albany during the session of the legislature.

The nominating committee presented its work in a very complete manner. Dr. James D. Spencer, of Watertown, was chosen as president, Dr. L. D. Bulkley, of New York, vice-president; Dr. F. C. Curtis, of Albany, secretary, and Dr. C. H. Porter, of Albany, Treasurer. The usual standing committees were elected, and delegates were sent to other state societies and associations. Dr. H. R. Hopkins, of Buffalo, was chairman of the business committee and performed his work in a most satisfactory manner. The fact is apparent always, that in this society much depends upon the efficiency of the business committee for the success of its meetings.

The prize essay committee awarded a prize of $100 to Dr. A. L. Benedict, of Buffalo, for an essay on Neuroses of the Stomach. Dr. Benedict took the prize last year on an allied topic.

